# The Core of Caring: Human Dignity in Nursing Through a Constructivist Grounded Theory

**DOI:** 10.1111/scs.70222

**Published:** 2026-03-16

**Authors:** Duygu Yıldırım, Esra Akın

**Affiliations:** ^1^ Department of Nursing, Faculty of Health Science İzmir Katip Çelebi University İzmir Turkey

**Keywords:** dignity, grounded theory, human dignity, nursing, nursing care, nursing theory

## Abstract

**Aim:**

To examine the experiences of nurses and academician nurses and develop a nursing theory that describes the processes of their perception of human dignity in their nursing care.

**Methods:**

A qualitative study guided by the constructivist grounded theory approach. Between May 2023 and February 2024, observations and semi‐structured interviews were conducted with 20 nurses and nurse academics. Data were analysed using constructivist grounded theory with three‐phase coding (open, focused, theoretical) and supplemented by situational analysis mapping to capture contextual factors in nursing care.

**Results:**

Four theoretical codes were identified—definition of dignity, being dignified, dignity violation and dignified care—derived from 14 focused codes.

**Conclusion:**

Human dignity is a central principle of nursing care, shaping actions and outcomes. Integrating this model into nursing education and practice may enhance patient‐centred care, strengthen professional identity and improve overall care quality. Future research should examine its impact on patient experiences and clinical outcomes.

## 1 Introduction

Dignity is a fundamental concept in nursing care, carrying critical importance for both patients and nurses. It extends beyond clinical procedures to encompass respect, compassion and recognition of individuals' intrinsic worth, making it central to the ethical responsibilities and professional roles of nurses [1]. Preserving dignity in care requires acknowledging the uniqueness and inherent value of each person, positioning nurses as key agents responsible for safeguarding this fundamental human right [2, 3].

Nurses' ethical attitudes and professional values play a pivotal role in preventing violations of patients' dignity [3, 4]. Yet, despite its ethical importance, dignity remains an abstract and ambiguous concept in nursing literature. The lack of conceptual clarity hinders the establishment of a shared understanding of how dignity should be defined and applied in clinical practice. Previous studies emphasise nurses' obligation to uphold patients' dignity, highlighting that this responsibility is shaped by both external ethical norms and internalised professional values [5, 6, 7]. Importantly, nurses' perceptions of dignity extend beyond recognising intrinsic human worth. They also encompass the ethical duties and responsibilities that shape professional practice. Dignity directly influences the quality of care delivered and profoundly affects patient experiences within nurse–patient interactions [8, 9]. Thus, it is essential to examine how nurses' ethical attitudes contribute to the preservation of patient dignity and how these attitudes are reflected in everyday care practices [10].

A review of the existing literature shows the absence of a comprehensive conceptual model explaining how dignity can be understood, defined and operationalised in nursing care. To address this gap, the present study employs a constructivist grounded theory approach to explore the core concepts, values and processes involved in delivering dignified nursing care.

## 2 Background

Dignity is widely regarded as a fundamental human value, defined as the intrinsic respect an individual holds for their existence and identity [5]. In theoretical discourse, dignity is often described through two interrelated dimensions: inherent dignity—the unconditional worth attributed to all individuals by virtue of being human, and acquired dignity—which is shaped by social context, behaviour and lived experience [11]. Historically, dignity has been framed from three major perspectives: theology, which views dignity as deriving from humans being created in the image of God; social hierarchy, where dignity is relative to position, rights and duties; and Kantian ethics, which anchors dignity in rational agency and autonomous moral decision‐making [5, 12, 13, 14]. Over time, dignity has been explored across multiple disciplines, including theology, philosophy, political theory, law, medicine and nursing [1, 2, 3]. Despite this interdisciplinary attention, it remains conceptually vague and inconsistently applied. Multiple terms, such as *b* *asic dignity, personal dignity, social dignity* and *human dignity*, are used without a shared definitional framework [12, 15]. This lack of clarity is especially problematic in nursing practice, where consistent ethical guidance is essential.

In healthcare, the central focus is the human being and the preservation of life. Nurses, as primary care providers, are tasked not only with clinical responsibilities but also with upholding ethical standards. International organisations such as the American Nurses Association (ANA) and the International Council of Nurses (ICN) recognise dignity as a core ethical value, while the American Association of Colleges of Nursing (AACN) defines it as respect for the inherent worth and uniqueness of individuals and communities [5]. The literature distinguishes between *preservation of human dignity*, respect, autonomy and individualised care, and *preservation of dignity in care*, supporting self, hope, pride, resilience and spiritual integrity within interactions [4, 16]. Together, these dimensions demonstrate that dignity influences not only *what* care is provided, but also *how* it is delivered [17].

Dignity‐centred care yields tangible benefits: patients experience value, respect and greater engagement; nurses report enhanced professional satisfaction; and institutions benefit from improved trust, reputation and overall care quality [18, 19]. Despite these positive outcomes, the literature lacks a clear conceptual model to guide dignity‐centred nursing care. This absence hinders nurses' ability to systematically integrate ethical values into practice, limiting the effectiveness of holistic care.

In today's complex and dynamic healthcare environment, ethical reflection requires nurses to ask not only ‘What can I do?’ but also ‘What should I do?’ [3]. Addressing dignity within this framework demands empirical investigation of how it is interpreted and enacted in practice. Qualitative research, especially constructivist grounded theory, is well suited to uncovering the meaning, complexity and lived experience of abstract concepts like dignity [20].

Given the scarcity of theoretical frameworks in this area, this study employs constructivist grounded theory to explore the core components of dignity in nursing care and to develop a conceptual model that bridges theory and practice. This model aims to support nurses in systematically applying ethical values, enhancing patient‐centred care and strengthening both clinical and organisational outcomes.

## 3 The Study

### 3.1 Aim, Research Question

The aim of this study was to develop a nursing theory that explains the experiences of clinical nurses and nurse academics regarding the meaning and processes of dignity‐centred nursing care. Specifically, the study sought to construct a theoretical model that illustrates how dignity is conceptualised, operationalised and integrated into nursing practice and education.

Based on this aim, the following research questions guided the study:

1. What are the key phenomena, concepts and processes that shape dignity‐centred nursing care?

## 4 Methods

### 4.1 Study Design and Theoretical Framework

This study adopted Charmaz's constructivist grounded theory (GT) methodology, which provides a systematic yet flexible approach for developing theoretical insights into complex and abstract phenomena. Constructivist GT emphasises the co‐construction of meaning between researchers and participants, enabling the development of interpretive theoretical models that move beyond description to generate explanatory insights [21, 22].

Given the multifaceted and context‐dependent nature of human dignity in nursing care, this approach was considered particularly suitable for exploring how nurses conceptualise, interpret and integrate dignity into their practice. Constructivist GT facilitated an in‐depth examination of participants' experiences and meaning‐making processes while accounting for the influence of social context and professional interactions [22].

The study was conducted in a university and a hospital, Turkey. This setting provided access to both clinical nurses and nurse academics, ensuring variation in experiences and perspectives regarding dignity in care [21].

### 4.2 Sampling Strategy and Participant Recruitment

In qualitative research, where fixed sample sizes are not predetermined, the adequacy of sampling is guided by the principle of theoretical saturation. In this study, purposive sampling with maximum variation was initially employed to capture a wide range of experiences, consistent with the principles of theoretical sampling. Theoretical saturation, which underpins this approach, is achieved when data collection yields no new themes or concepts and the information becomes repetitive—signalling both sufficient breadth and depth [21].

Participant recruitment was conducted in two sequential stages to ensure methodological rigour. In the first stage, criterion‐based purposive sampling was applied; participants were selected according to predefined inclusion criteria (Table 1). In the second stage, theoretical sampling was undertaken. Emerging codes and categories from the initial analysis guided the recruitment of additional participants, allowing for the elaboration, refinement and contrast of concepts [23, 24]. In total, 20 participants were recruited: three clinical nurses and three nurse academics in the first stage, followed by 14 additional participants (nurses and nurse academics) in the second stage. Data collection was discontinued when theoretical saturation was achieved—that is, when interviews produced no novel insights and responses became repetitive.

**TABLE 1:** Inclusion criteria of nurses and nurse academics.

Criteria	Inclusion of nurses	Inclusion of nurse academics
Gender	Male and female	Male and female
Participants	Working in internal medicine, surgical clinics, intensive care clinics and outpatient treatment centres	Working in university department of nursing
Degree	Minimum a bachelor's degree	Doctorate degree
Work experience	Minimum of 1 year in the clinic where he/she works	Have at least 1 year of clinical experience
Consent	Agreed to participate in interviews/observations and use of audio recorders	Agreed to participate in interviews/observations and use of audio recorders

### 4.3 Data Collection

Data were collected through face‐to‐face, in‐depth individual interviews conducted between May 2023 and February 2024. Two primary instruments were used: an individual demographic form and a semi‐structured interview guide.

The demographic form collected information on nurse participants' age, gender, professional title, educational background, year of graduation, current clinical unit, institutional role and total duration in that profession. For academic nurses, additional information regarding clinical experience and academic position was obtained.

The semi‐structured interview guide, developed by the researchers, initially included six open‐ended questions to explore participants' perspectives on human dignity in nursing. Sample questions included: *What is a human being?*, *What is dignity?*, *What is human dignity?*, *Who is a dignified person?*, *Can human dignity be violated?* and *What does human dignity mean to you in nursing practice?* As interviews progressed, the guide was adapted to incorporate follow‐up questions emerging from prior responses, such as: *What factors influence the loss or enhancement of dignity?*, *Is the concept of human dignity significant in nursing care?* and *What actions are taken to preserve human dignity in practice?*

All interviews were conducted in Turkish and audio‐recorded with participants' consent. They took place in quiet, mutually agreed‐upon locations, such as private rooms in hospitals or faculty meeting rooms, to ensure participants' comfort and minimise interruptions. Interviews lasted approximately 45–90 min. All individual interviews were conducted by a single researcher to prevent power imbalances and to ensure that the sessions were conducted in an equal, consistent and unbiased manner. Throughout the study, the researcher took detailed notes during each interview and maintained analytic memos. These memos were used to enhance analytical depth, guide the analysis, clarify emerging themes, resolve uncertainties and support reflexivity in line with GT principles. The memos played a key role in shaping the direction of some themes and in the development of the theoretical model. Audio recordings were transcribed verbatim, and the transcripts were reviewed to ensure accuracy and to capture underlying meanings. Any transcription errors were corrected. Data collection continued until no new themes or concepts emerged, achieving theoretical saturation.

### 4.4 Data Analysis

**FIGURE 1:**
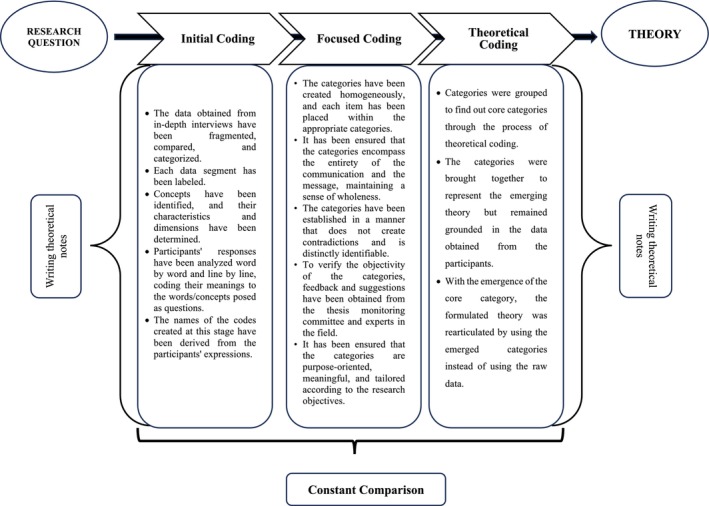
Coding and analysis process in constructivist grounded theory.

In constructivist grounded theory studies, data collection is followed by iterative data analysis, and this cyclical process continues until a comprehensive theoretical understanding is achieved [22]. Accordingly, this study adhered to the grounded theory principle of initiating data analysis early, immediately after collecting a small portion of data. Data analysis was conducted using Charmaz's three‐stage coding process: initial coding, focused coding and theoretical coding. Throughout the analysis phase, the constant comparative method was employed, enabling simultaneous data collection and analysis (Figure 1).

During the initial coding phase, interview transcripts were coded line‐by‐line, paying close attention to nuances, explicit statements and implicit meanings. The first three interviews were coded and analysed prior to further data collection. This iterative process of simultaneous data collection and analysis continued until theoretical saturation was reached. In total, 371 initial codes were generated. In the focused coding phase, these initial codes were grouped into broader categories. The categories were continuously reviewed and refined, progressively moving toward more abstract conceptualisations. This phase concluded when theoretical saturation was achieved, indicated by no emergence of new properties or theoretical insights within the categories. Ultimately, 14 focused categories were identified. The final stage, theoretical coding, involved synthesising the focused categories to develop a coherent GT. Categories were grouped to identify core categories that represent the central phenomena of the study. This process ensured that the emerging theory remained firmly grounded in participants' data while providing conceptual clarity. With the identification of the core category, the theory was rearticulated using these categories rather than raw data. As a result, four theoretical codes were established, forming the foundation of the study's substantive theory.

### 4.5 Ethical Considerations

Ethical approval for this study was obtained from the Izmir Katip Çelebi University Social Research Ethics Committee (Approval Number: 2023/09‐01, April 2023) and the Izmir Provincial Directorate of Health (Approval Number: 77597247/354). Institutional permissions were also granted by the university's nursing faculty and the affiliated hospital where the research was conducted.

Participants were fully informed about the voluntary nature of their participation, the confidentiality of the interviews and their right to withdraw at any time without consequence. To protect participants' identities, self‐selected pseudonyms were used in transcripts, translations and quotations. Verbal informed consent was obtained from all participants prior to the commencement of each interview. All interview recordings, transcripts and analytic documents were stored in password‐protected folders on secure devices, with access restricted to the researchers.

### 4.6 Rigour

In qualitative research, validity pertains to the accuracy and truthfulness of findings, while reliability refers to the consistency and replicability of results. In GT studies, addressing both validity and reliability is essential to establish methodological rigour. In this study, credibility and transferability were applied to ensure validity, whereas confirmability and dependability were applied to ensure reliability [25, 26].

To enhance credibility, the research team conducted regular peer debriefings to analyse data, review transcripts and discuss emerging findings. Reflexivity was maintained throughout the study by documenting reflective memos addressing potential biases, assumptions and methodological decisions. To improve transferability, detailed descriptions of the research design, study sample, data collection instruments and procedures, data analysis methods and theoretical generalisations were provided. These descriptions were also compared with relevant literature to demonstrate the applicability of findings beyond the immediate context. Theoretical sampling was employed to capture a broad range of experiences, including perspectives from both clinical nurses and academic nurse academics.

Confirmability was ensured through collaborative content analysis between the researcher and the advisor using an inductive approach for code development. A kappa analysis was performed to assess inter‐coder agreement, resulting in a kappa value of 0.85, indicating excellent reliability. The finalised coding framework was also reviewed by an external nursing expert to ensure its robustness. Dependability was addressed by maintaining a research diary throughout the study to document methodological decisions and reflections. Direct quotations from participant interviews were included to preserve data authenticity and to strengthen the credibility of findings by aligning them with emergent concepts and themes.

## 5 Results

The majority of participants were aged 26–35 years (35%, *n* = 7) and 36–45 years (35%, *n* = 7), while a smaller proportion were aged 46–55 years (30%, *n* = 6). The mean (SD) age of participants was 39.45 ± 9.62 (range: 26–55) years. The mean (SD) years of work experience was 16.9 ± 9.63 years. Detailed characteristics of the participants are presented in Table 2.

**TABLE 2:** Demographic and professional characteristics of the participants.

Participant code	Role	Age	Gender	Education level	Graduation year	Unit/department	Total work experience
P1	Nurse	33	Female	MSc	BSc: 2013 MSc: 2023	Obstetrics Clinic	10 years
P2	Nurse	43	Female	MSc	BSc: 2006 MSc: 2021	Cardiovascular Surgery Clinic	22 years
P3	Nurse	27	Female	BSc	BSc: 2018	Medical Oncology Unit	5.5 years
P4	Nurse Academic	48	Female	PhD	BSc: 1995 MSc: 2006 PhD: 2011	Department of Women's Health and Diseases	27.5 years
P5	Nurse Academic	50	Female	PhD	BSc: 1994 MSc: 1999 PhD: 2008	Department of Nursing Education	29 years
P6	Nurse Academic	48	Female	PhD	BSc: 1997 MSc: 2003 PhD: 2010	Department of Nursing Education	26 years
P7	Nurse	37	Female	MSc	BSc: 2008 MSc: 2011	Palliative Care in Cancer Patients Clinic	15 years
P8	Nurse	31	Female	BSc	BSc: 2015	Cardiology Angio Clinic	8 years
P9	Nurse	43	Female	BSc	BSc: 2002	Cardiology Angio Clinic	22 years
P10	Nurse Academic	52	Female	PhD	BSc: 1993 MSc: 2003 PhD: 2007	Department of Internal Medicine Nursing	31 years
P11	Nurse Academic	55	Female	PhD	BSc: 1993 MSc: 2007 PhD: 2012	Department of Mental Health Nursing	26 years
P12	Nurse Academic	52	Female	PhD	BSc: 1992 MSc: 1996 PhD: 2003	Department of Women's Health Nursing	32 years
P13	Nurse Academic	43	Female	PhD	BSc: 2003 MSc: 2008 PhD: 2013	Department of Internal Medicine Nursing	17 years
P14	Nurse Academic	40	Female	PhD	BSc: 2006 MSc: 2009 PhD: 2015	Department of Public Health Nursing	18 years
P15	Nurse	27	Female	BSc	BSc: 2018	General Intensive Care Unit	6 years
P16	Nurse	27	Female	MSc	BSc: 2018 MSc: 2022	Anesthesiology & Reanimation Intensive Care Unit	5 years
P17	Nurse	28	Female	BSc	BSc: 2019	Pulmonology Clinic	4 years
P18	Nurse Academic	42	Female	PhD	BSc: 2003 MSc: 2006 PhD: 2012	Department of Public Health Nursing	15 years
P19	Nurse Academic	37	Female	PhD	BSc: 2008 MSc: 2011 PhD: 2017	Department of Internal Medicine Nursing	15 years
P20	Nurse	26	Male	MSc	BSc: 2019 MSc: 2023	Cardiology Clinic	4 years

The research findings are initially presented under the theoretical code ‘Definition of Dignity’, which outlines the conceptual structure of the notion of dignity. Data related to the focused codes are then organised under thematic headings within the theoretical codes ‘Being Dignified’ and ‘Violation of Dignity’. Following the explanation of the core components of the Human Dignity Model in nursing care, the theoretical code ‘Dignified Care’ is introduced. Within this framework, the concepts of ‘Facilitators of Dignified Care’, ‘Dignified Care Actions’ and ‘Barriers to Dignified Care’ are discussed from a conceptual perspective. Participant statements related to each code are presented as direct quotations to illustrate the findings.

### 5.1 Theoretical Code 1: Definition of Dignity

In the study, participants defined dignity within a broad framework, conceptualised under six dimensions: being deserved through the capacity to think, value judgement, giving/receiving respect, being valued, dignity as an emotion and being inherently present from birth (Table 3). Participants emphasised that defining humans by their capacity to think positions this trait as the source of fundamental rights, and that dignity constitutes a universal human right enabling individuals to exercise these rights freely and equally.

…When I think of human dignity, I see the two concepts as inseparable. Dignity alone does not have meaning; it is inherently part of being human. Whether someone works or not, as long as we live on this earth and can think, I believe everyone deserves dignity. It also feels like an internal drive, something that exists within us. (P2, Female)

… Protection of our rights, being recognized as individuals, and not having our freedom restricted in society. (…) When I think of human dignity, it means having the right to freedom, exercising these rights as an individual, and using the freedom granted by one's personality. (P7, Female)

Some participants described humans as moral, ethical and spiritual beings whose values shape life. Dignity was often regarded as overarching and inclusive, and could be defined either in terms of avoiding negative experiences such as humiliation or in terms of positive experiences such as trust, recognition and acceptance.

…Dignity is a value judgment in which a person sets limits on what they cannot accept, based on their own sense of values and identity. It is a unique value system of the individual. (P12, Female)

…Dignity is when an individual can live without being humiliated by others and can satisfy basic needs, such as having enough food, without being subjected to minor degrading situations throughout life (…) In other words, dignity can be considered as living a life with respect and self‐worth. (P1, Female)

Participants also linked dignity to self‐respect, esteem, respect for others and the life cycle of all living beings. Some described it as being valued and recognised by others, while others emphasised dignity as an innate quality present from birth, bestowed by God and carrying intrinsic motivational significance.

(…) Beyond basic, hedonistic needs, dignity involves including respect within life, incorporating empathy (…) It is about respecting the entire cycle of life that exists in wholeness. Dignity applies to a person, to a living being, to a flower, and even when interacting with an animal. This is how I interpret dignity. (P10, Female)

…So, I think it is something that exists within all of us, instinctual, given by God. Just as we have physiological and spiritual elements that sustain our metabolism, dignity is similarly an inherent quality. (P2, Female)

**TABLE 3:** Coding Framework of dignity and dignified care: A grounded theory approach.

Theoretical code	Focused code	Open/initial code
Definition of dignity	Value from cognitive capacity	—
	Value judgement	
	Dignity as an emotional experience	
	To be valued	
	Showing respect/Being respected	
	Inherent in the individual	
Being dignified	Being a dignified individual	Having self‐awareness Being self‐confident Being aware of one's rights Possessing moral values Social acceptance Making informed decisions Demonstrating moral behaviour
	Factors promoting dignified behaviour	Fulfilment of basic needs Family teachings Personal development process Social perspectives Living environment
	Enhancement and reinforcement of dignity	Individual factors Social factors
	Rejection of societal labelling of the individual as undignified	
Violation of dignity	Ignore	Ignoring the individual Disregarding opinions Disregarding emotions
	Deprivation	Inadequate communication/information Deprivation of needs
	Invasion of personal space	—
	Oppressive use of power	
	Paternalistic behaviours	
	Violation of physical and psychological integrity	
	Undermining one's sense of worth	
Dignified care	Orientation toward dignified care	Awareness of providing human care Demonstrating empathy Acknowledgment of rights Professional sensitivity
	Dignified care actions	Being responsive to needs Respect for decisions and choices Protection of privacy Acceptance of cultural and spiritual characteristics Interactive communication Fulfilling the need for information and being informed Ensuring equality through justice Avoidance of materialistic approach Ensuring information security Protective behaviour against harm
	Barriers to dignified care	Questioning one's existence Rejection of professional ıdentity
	Outcomes of dignified care	Adherence to care and treatment Professional satisfaction

### 5.2 Theoretical Code 2: Being Dignified

The theoretical code ‘being dignified’ comprised four focus codes: ‘being a dignified individual’, ‘factors promoting dignity’, ‘strengthening/supporting dignity’ and ‘rejection of the concept of an undignified individual’ (Table 3). Participants described a dignified individual as self‐aware, confident, rights‐conscious and guided by moral values, with dignity reinforced through honesty, compassion and respect for oneself and others.

… A person with a sense of dignity is confident in their steps. They can act without hesitation, regret, fear, or anxiety in what they do (…). (P11, Female)

Meeting basic needs was considered essential, alongside family support, education, personal development and societal recognition. Both individual factors (e.g., self‐discovery, achievement, self‐actualisation) and societal factors (e.g., honouring actions, appreciation, acknowledgement) were reported to strengthen dignity.

… Human dignity develops from the moment a child is born through education. One does not simply claim, ‘I am dignified at this age’ and have it accepted; rather, others observe one's behaviors. A child grows and learns by observing their environment and receiving guidance. If a child's wrong actions are reinforced, they internalize that behavior. Therefore, it is important to nurture and guide the child correctly from a young age. I believe the roots of dignity start forming at this stage. The education of dignified individuals begins in the family and continues through school—primary and secondary education. (P5, Female)

Participants emphasised that dignity is innate and rejected the notion of an undignified individual. Perceptions of undignified behaviour were understood as temporary disruptions shaped by others' judgements, rather than the absence of dignity.

There is no individual without dignity. Each person behaves according to their own values. It is only when their values conflict with someone else's that this may be perceived differently (…). Others may view it this way when interests clash (…). This perception arises when a person's values are not recognized or accepted. (P18, Female)

### 5.3 Theoretical Code 3: Violation of Dignity

Participants described dignity violation as the neglect, disregard or devaluation of individuals' opinions, feelings and values, which can occur in social, professional and personal contexts. Key manifestations included deprivation of information and essential needs, intrusion into personal space, misuse of power, paternalistic behaviours, interference with physical and psychological integrity, and inadequate recognition of competence (Table 3).

(…) For example, if you do not acknowledge someone's existence, you avoid eye contact. You do not want to see them; you do not recognize their presence (…). You ignore eye contact, devalue their opinions, and strip the person of identity. For instance, in a hospital, you might refer to them by room number or bed number (…). (P11, Female)

Participants emphasised that such behaviours threaten emotional, physical and social integrity, highlighting that communication, respect for autonomy and acknowledgment of personal and professional contributions are central to protecting and promoting dignity in care contexts.

(…) For example, checking a patient's blood pressure without looking at them, simply measuring it and leaving, or saying ‘extend your arm, I will take blood’ without explaining the purpose or engaging in any dialogue, and performing the procedure without interaction, can be considered behaviors that undermine the patient's dignity. (P 1, Female)

… Dignity can be compromised when one's personal traits are attacked or when one is belittled. Material possessions may matter, but I believe the greatest threat to dignity occurs when a person's personal characteristics are targeted. (P19, Female)

### 5.4 Theoretical Code 4: Dignified Care

Within the theoretical code ‘Dignified Care’, four focused codes were identified: ‘orientation toward dignified care’, ‘dignified care actions’, ‘barriers to dignified care’ and ‘outcomes of dignified care’ (Table 3). These subcodes structure the presentation of participants' perspectives on how dignity is preserved or compromised in nursing care.

The study findings indicate that nurses' orientation toward dignified care is guided by awareness of patients as human beings, empathy, recognition of patients' rights and professional responsibility. Participants emphasised sensitivity to vulnerability, respect for dignity throughout care and acknowledging the patient's presence. Empathy, described as ‘putting oneself in the patient's place’, was identified as a central motivator, fostering understanding of emotional needs, respect for privacy and protection of others' dignity, which also strengthens the nurse's own sense of dignity. Participants stressed that patients inherently deserve dignified care; healthcare professionals should avoid paternalistic approaches, and informed decision‐making, equitable care and professional sensitivity are fundamental to delivering respectful and compassionate nursing practice.

… My approach toward others should be based on acknowledging that they are human beings, individuals, a mother, a spouse, a father, or a child. (P5, Female)

… In the nursing profession, I believe that humans are the most valuable beings. For example, when providing care, I truly give care as if I were caring for myself or a loved one, even though this may sound like a cliché. (P6, Female)

Dignified care actions included responsiveness to needs, respect for decisions, preservation of bodily integrity, recognition of cultural and spiritual characteristics, interactive communication, information provision, fairness and protection from harm. Privacy, informed consent and autonomy were central to care, particularly in end‐of‐life situations. Effective communication enhanced trust, patient motivation and care quality.

(…) Sometimes, situations can be resolved with a stent, especially in coronary artery patients. In such cases, we discuss that the patient should evaluate the options and make decisions themselves, rather than having a choice made by someone else. These are important for dignity because they concern the individual's own body. (P2, Female)

Privacy is not just about covering a person's exposed areas; every individual has a personal energy field that should not be invaded. If the person does not want others to enter this space, respect should be shown, and one should remain outside (…). Any intervention performed without consent in such cases constitutes a violation of privacy. (P12, Female)

Barriers to dignified care were identified as workplace stressors, including mobbing, high workload, long working hours, time pressures, burnout and lack of financial satisfaction. These factors were reported to impede holistic care, reduce motivation and limit nurses' ability to advocate for patients, creating dilemmas between safeguarding their own values and protecting patient dignity.

… I can mention two things: both unawareness and the intensity of work. Of course, ideally, we would work under better conditions with fewer patients per nurse. However, when this is not possible, the workload can take over, and I believe it can lead to overlooking the patient's dignity, which I have observed happening. (P17, Female)

Dignified care was reported to enhance patient well‐being, treatment adherence and nurse–patient relationships, while supporting nurses' emotional fulfilment and professional satisfaction.

In patient care, nurses should act in ways that honor human dignity. This includes respecting patients' privacy, behaving accordingly, and creating an environment of trust. Conducting care within the framework of mutual respect and compassion, and being aware of patients' rights and human rights (…), allows us to discharge patients more happily, healthier, and better recovered. (P20, Male)

… Sometimes, it may involve simply talking, having someone listen, or providing comfort. Therefore, care is not only about administering medication or treatment but also about the emotional aspects—mutual interaction, communication, and engagement each gain importance. Even holding a patient's hand and saying, ‘Thank you’ creates mutual satisfaction. Expressions of gratitude were among the factors that most contributed to professional fulfillment. (P9, Female)

### 5.5 Human Dignity in Nursing Care: A Data‐Driven Theoretical Model

In this study, participants described the concept of dignity using expressions such as the capacity to think, emotion, being valued, moral judgement and an innate quality. These definitions were shaped within the framework of respect. The development of a dignified individual depends on multiple factors, primarily on the person's awareness of the concept of dignity. An individual who understands the notion of dignity is considered to be aware of their worth, capable of sensing it and recognising it as a right. Moreover, awareness and respect for one's emotions and needs contribute to the development of self‐confidence, and a dignified individual is often described as a self‐confident one. Being valued also emerges in relation to societal perceptions. The foundation of being seen as a dignified individual lies in how society perceives and treats the person. Individuals who are accepted by society were often referred to by participants as possessing dignity. Furthermore, a person who acknowledges dignity as a right and behaves in accordance with moral norms and ethical consciousness is regarded as dignified. In addition to these, family teachings and the individual's personal developmental trajectory play a crucial role in the formation and evolution of dignity. A person who experiences dignity as an innate quality through family education or recognises it during their personal development is also viewed as a dignified individual. In the context of nursing care, a person who defines dignity is one who has discovered their own sense of dignity. This awareness enables the individual to recognise that they are caring for another human being, fosters empathy and directs them toward providing dignified care with professional sensitivity while fully acknowledging patients' rights. Within this framework, dignified care practices are shaped by the following antecedents: ensuring equity and justice in care for all patients, avoiding materialistic approaches, demonstrating sensitivity to all patient needs, engaging in interactive communication, respecting patients' cultural and spiritual characteristics during care, involving them in decision‐making, ensuring access to information and protecting their right to privacy.

**FIGURE 2:**
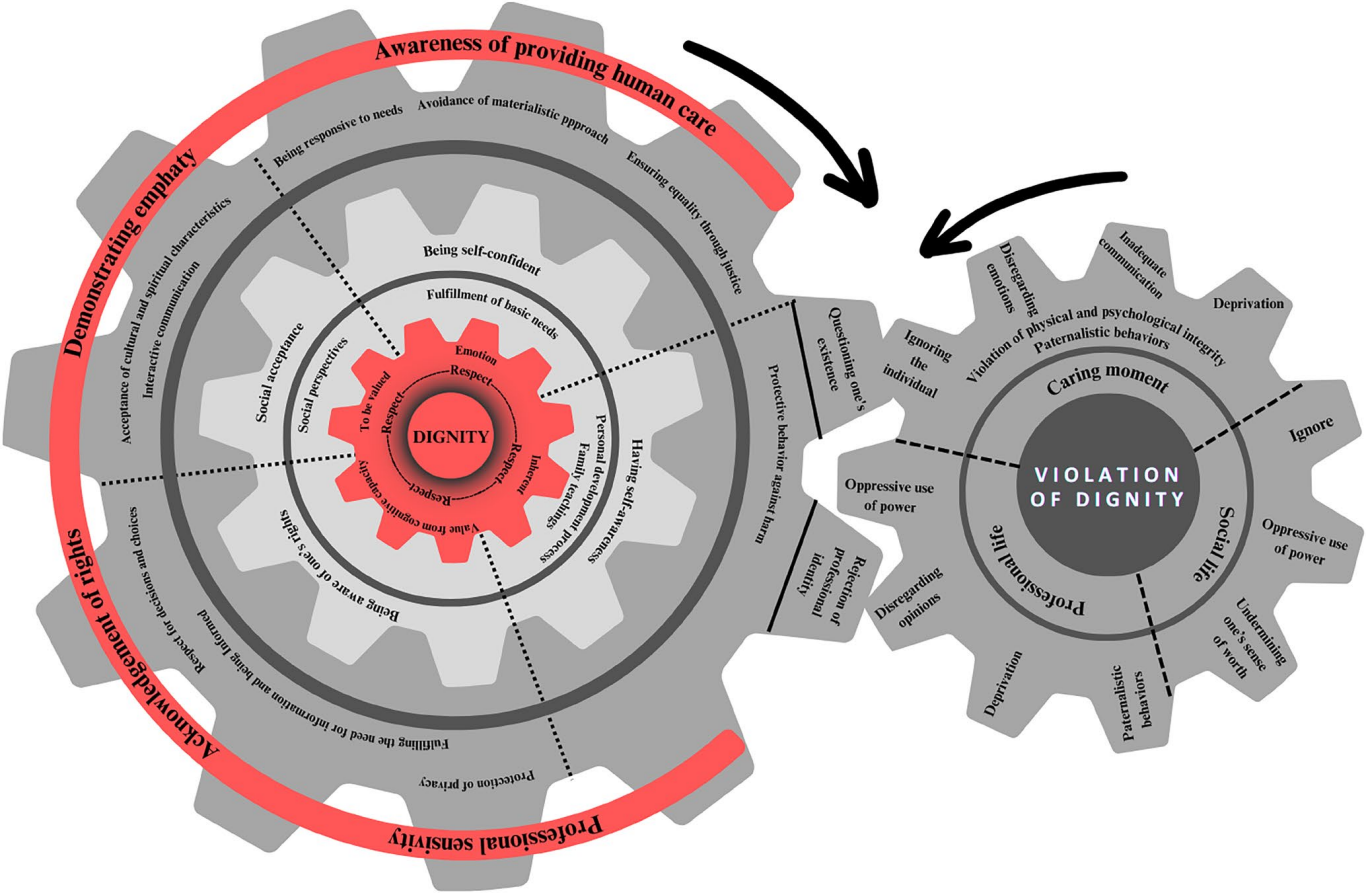
Human dignity in nursing care: A data‐driven theoretical model.

Nevertheless, certain factors significantly hinder the implementation of dignified care practices. These include nurses questioning their existence and the denial of their professional identity. The findings of this study revealed that obstacles to dignified care practices are closely linked to violations of dignity. These violations manifest across three primary contexts: professional life, social life and the moment of care. Violations during the care process include ignoring the individual, neglecting their emotions, insufficient communication, paternalistic behaviour, interference with physical and psychological integrity, and deprivation. In the social sphere, dignity violations also stem from being ignored, starting with disregarding one's presence in life, extending to the use of power against the individual, and devaluation of the person or their actions. Finally, violations of dignity can also occur within professional life, where individuals may experience being ignored through the dismissal of their ideas, exposure to paternalistic behaviour, misuse of professional power or deprivation from essential resources or opportunities. In light of these findings, the ‘Human Dignity in Nursing Care Model’, a data‐driven theoretical framework, proposes that dignified existence and dignified care practices move in the same direction, with dignity at the core. All concepts, factors and processes are interlinked through permeable, mutually influential connections. However, factors and processes that interrupt dignified care create impermeable, continuous barriers, giving rise to the cycle of dignity violations. The interconnectedness of concepts, factors and processes within the cycle of dignity violations implies that violations occurring in professional life, social life and care contexts mutually influence one another. The cycle of dignity violations operates independently of the dignity core, moving in the opposite direction of the dignity wheel (Figure 2).

## 6 Discussion

This study explored nurses' and nurse academics' perspectives on dignity through four interconnected themes: the definition of dignity, being dignified and factors promoting dignity, situations and actions perceived as violations of dignity, and dignified care practices, including facilitating and hindering factors.

The literature reveals that dignity remains a concept with no universally accepted definition, often described as complex, multidimensional and ambiguous [12]. Across disciplines such as philosophy, theology, law, sociology, medicine and nursing, dignity is regarded as a central concept with both theoretical and practical significance, particularly in the contexts of human rights, healthcare and social justice [15]. In nursing practice, having a conceptually clear understanding of dignity is essential for its effective translation into clinical practice. In our study, participants defined dignity as an inherent, relational and ethically grounded attribute of human beings. Supporting this conceptualisation, Delmar's relational nursing ethics perspective emphasises that human beings are interdependent and that care takes place within networks of trust and relational obligations [27]. Participants also associated dignity with capacities such as reasoning, moral discernment and empathy, an approach consistent with Confucianism, Kantian ethics and contemporary conceptualisations [2, 14]. Moreover, in our study, dignity emerged as a multidimensional concept encompassing moral, ethical and spiritual values such as truthfulness, compassion and honesty, as well as emotional dimensions characterised by freedom from humiliation, oppression or degradation. Therefore, dignity should be understood not only as an individual attribute but also as a relational experience shaped by interactions with others and by whether an individual's value is recognised or denied.

When examining factors that support being dignified, participants drew attention to the relational and context‐specific nature of dignity. According to Delmar, within Martinsen's ethics of care approach, individuals' autonomy and scope for action develop through mutual interactions. These interactions create an environment in which patients' ability to express themselves and their scope for action may either expand or be restricted [27]. In this context, awareness of oneself, others and the environment, along with the internalisation of moral and social values, plays a central role in the development and maintenance of dignity. Family teaching, educational experiences, professional responsibilities and societal recognition strengthen individuals' understanding of dignity by supporting both internal awareness and relational expressions of respect [28]. In addition, society's perspective toward the individual also guides the individual toward being dignified in a relational sense. This implies that when society helps individuals recognise their inherent sense of worth, they are more likely to orient themselves toward dignity. Individuals who strive to be dignified may outwardly reflect this orientation through valuing themselves and others, self‐belief, a sense of competence, independence and freedom of choice [29]. In this regard, our findings demonstrate that a dignified individual exhibits self‐confidence, moral and ethical awareness, social acceptance, empathy and responsibility toward others. Furthermore, participants emphasised that dignity persists even in the absence of external recognition, highlighting its inalienable nature and reinforcing the view that violating others' dignity simultaneously diminishes one's own dignity [30].

Nevertheless, every human interaction carries the potential to become a dignified encounter, one in which dignity may either be upheld or violated. Such encounters involve individuals and relationships [29]. Our study revealed that ignoring individuals, denying their participation in decision‐making processes, failing to provide adequate information, violating personal space, exercising authority in a paternalistic manner, and devaluing opinions pose serious threats to dignity. These findings underscore the concept of power asymmetry in healthcare settings [30, 31]. In the nurse–patient relationship, power is inherently asymmetrical; patients are dependent on nurses due to health conditions beyond their control, and the balance of power often tilts in favour of the nurse. This asymmetrical power structure emerges as a fundamental factor directly influencing both the formation and preservation of trust. Although trust may be given as a natural aspect of life, it remains vulnerable and can be undermined by nurses' attitudes and actions. An accepting and sensitive nursing approach expands patients' scope for action and reinforces trust, whereas rejecting, oppressive or overly controlling behaviours restrict patients' autonomy and participation, placing their dignity and personal integrity at risk. Therefore, nurses' ethical responsibility lies in being aware of their use of power and in providing relationship‐sensitive, humane care that protects patients' scope for action. At this point, focusing on nurses' roles and behaviours in daily care practices is critical for understanding the effects of power asymmetry on patients [27, 30, 32]. Based on our findings, it can be reasonably stated that nursing care should involve respect for patient autonomy, protection of privacy and active participation in care‐related decisions, all of which play an important role in preventing dignity violations.

As participants orient themselves toward dignified care, they place dignified care actions at the centre of care and manage their practices accordingly [33]. In our study, dignified care practices were conceptualised as behaviours that operationalise the ethical principles of respect, autonomy and non‐maleficence. Participants identified sensitivity to patients' needs, protection of physical and psychological integrity, cultural and spiritual sensitivity, interactive communication, safeguarding information and prevention of harm as core components of dignified care. Previous studies similarly emphasise empathy, patient‐centred communication and recognition of patients' unique identities as central to dignity‐focused nursing practice [8, 34]. In our study, participants also noted that the sustainability of dignified care depends not only on individual nursing behaviours but also on institutional support. They stated that facilitating factors for dignity‐centred nursing care include professional sensitivity, ethical awareness, respect for patient rights and empathetic engagement, whereas barriers such as high workload, time constraints, inadequate communication, limited resources and the undervaluation of nurses by institutions hinder the continuity of dignified care. When adequate staffing, resources and an institutional culture that values ethical and patient‐centred practices are provided, nurses are able to deliver care more empathetically and effectively while sustaining their professional satisfaction [8, 35]. This creates a positive cycle for both care providers and care recipients. In this context, it would not be incorrect to state that dignity‐centred care holds a central place in ethical nursing practice and represents an indispensable factor in strengthening trust, respect and therapeutic relationships.

Participants reported that dignified care produces significant outcomes for both patients and nurses. These outcomes include strengthened nurse–patient trust; consequently, increased patient engagement, improved adherence to treatment and enhanced professional satisfaction. Particularly when considering that trust constitutes the foundation of the nurse–patient relationship and possesses a fragile nature, it is appropriate to emphasise that establishing and sustaining trust requires continuous ethical attentiveness and reciprocal interaction. Therefore, the most critical aspect lies in building relationships that allow trust to emerge and in preventing any disruption within the trust relationship [27, 32]. This can only be achieved by viewing and understanding individuals through a holistic perspective and being sensitive to their needs across all dimensions. Nurses' responsiveness to patient needs and their maintenance of open and respectful communication encourage active patient participation in care processes, thereby enhancing adherence to treatment and contributing to positive clinical outcomes [10, 36]. Participants emphasised that recognising patients as human beings, enabling informed decision‐making and providing individualised care not only preserve patients' dignity but also strengthen nurses' sense of professional purpose and emotional fulfilment.

Overall, this study underscores that dignity in nursing is a complex, multidimensional construct rooted in inherent human value, relational engagement and ethical practice. The findings highlight that dignity is both a personal attribute and a socially mediated experience, shaped by interactions, institutional structures and professional responsibilities. To operationalise dignity in clinical practice, nurses must integrate cognitive, emotional and ethical considerations, ensuring that care not only meets physical needs but also affirms the intrinsic worth of every individual. Future research should continue exploring context‐specific applications of dignity‐centred care, including interventions to address systemic barriers, enhance nurse empowerment and support sustainable ethical practice in diverse healthcare settings.

### 6.1 Recommendations

Based on the study findings, it is recommended that human dignity be systematically integrated into nursing education at both undergraduate and postgraduate levels, addressing both theoretical foundations and practical application. Curricula should foster dignity‐centred competencies such as empathy, ethical decision‐making, patient‐centred communication and sensitivity to cultural and spiritual values, while training programs further emphasise ethical awareness, reflective practice and the recognition of barriers to dignified care. In this respect, the theoretical model proposed in this study may serve as a guiding framework for embedding dignity‐oriented perspectives across undergraduate and postgraduate nursing curricula, orientation programs and continuing professional development activities.

Rather than functioning as standalone instructional content, the model may be operationalised through brief and structured learning strategies integrated into existing courses. Short reflective modules and guided reflective exercises can support learners in critically examining everyday clinical encounters in which dignity is preserved, threatened or restored. In addition, case‐based discussions and simulation‐based ethical scenarios—where dignity‐preserving and dignity‐compromising care behaviours are explicitly demonstrated, enacted and compared—may enhance ethical sensitivity, relational competencies and moral awareness among both nursing students and practicing nurses. Within this educational framework, the role modelling of dignity‐based care by educators during preclinical skills training and clinical teaching emerges as a particularly important pedagogical strategy. These teaching processes may be evaluated through structured assessment forms, performance‐based evaluation tools used in simulation settings, rubrics applied in case‐based ethical discussions and curriculum‐aligned ethical learning outcomes, thereby allowing the systematic examination of dignity‐related learning processes.

The application and evaluation of conceptual models of dignified care in clinical practice are likewise essential for optimising patient outcomes, enhancing professional satisfaction and reinforcing the ethical foundations of nursing. Importantly, the theoretical model presented in this study is not intended to function as a summative or outcome‐oriented measurement tool; rather, it offers an explanatory framework that supports the enhancement of ethical sensitivity and quality of care in both clinical and educational contexts. By rendering visible the relational and contextual factors that enable or constrain dignity‐based care, the model provides a conceptual foundation for future research examining ethically sensitive care environments. Within this framework, future studies may explore feasible and context‐sensitive indicators through which the model's concepts can be examined in practice. At this stage, future research is encouraged to focus on patient‐centred indicators in clinical settings, including dignity‐related feedback prompts, patient experience surveys, analyses of patient complaints and commendations, incident reports related to privacy or confidentiality, and observational assessments of care interactions that preserve dignity. From the perspective of nurses, studies may examine how dignity is understood, enacted and sustained in everyday care practices, as well as the conditions under which it is protected or placed at risk. In this context, the use of tools such as moral distress screenings, assessments of ethical awareness and attitudes, value clarification exercises and case‐based evaluations of responses to dignity‐threatening or dignity‐preserving situations may provide valuable insights into the practical dimensions of dignity‐oriented nursing care.

### 6.2 Limitations

Although this study provides an in‐depth theoretical understanding of human dignity in nursing care, several limitations should be acknowledged. First, the study was conducted with a relatively small sample of nurses and nurse academics from specific clinical and academic contexts, which may restrict the transferability of the findings to broader populations or different healthcare systems. Second, as inherent in all qualitative research, the results are context‐bound and shaped by the subjective perspectives of participants, requiring careful consideration when applying them to other settings. Third, the use of constructivist grounded theory, complemented by situational analysis mapping, allowed for a rich and nuanced exploration of dignity; however, these interpretive methods are influenced by the researchers' positionality, potentially shaping data interpretation. Finally, while theoretical saturation was achieved, the model developed in this study warrants further testing and refinement in diverse nursing contexts and with larger, heterogeneous samples to enhance its applicability and to evaluate its impact on nursing practice and patient care outcomes.

## 7 Conclusion

This study highlights human dignity as a central, inalienable and relational concept in nursing care, encompassing cognitive, moral, emotional and spiritual dimensions. A dignified individual demonstrates self‐awareness, ethical behaviour, rights consciousness, empathy and social responsibility. Dignity persists even without external recognition, emphasising its inherent nature. Dignity violations occur across social, professional, managerial and care settings, including neglect, deprivation, paternalism, intrusion, devaluation and denial of decision‐making. Conversely, dignity‐centred care—guided by empathy, ethical sensitivity, patient‐centred communication and respect for cultural and spiritual values—enhances patient engagement, treatment adherence, recovery and professional satisfaction.

## Author Contributions

Duygu Yıldırım: conceptualisation, methodology, software, visualisation, investigation, supervision, data curation, writing – original draft, writing – review and editing. Esra Akın: conceptualisation, methodology, writing – review and editing, data curation, writing – review and editing. All authors reviewed the manuscript.

## Funding

The authors have nothing to report.

## Ethics Statement

Ethical approval for this study was obtained from the Izmir Katip Çelebi University Social Research Ethics Committee (Approval Number: 2023/09‐01, April 2023) and the Izmir Provincial Directorate of Health (Approval Number: 77597247/354). Institutional permissions were also granted by the university's nursing faculty and the affiliated hospital where the research was conducted.

## Consent

Written and verbal informed consent was obtained from participants who participated in this study.

## Conflicts of Interest

The authors declare no conflicts of interest.

## Data Availability

The data that support the findings of this study are available on request from the corresponding author. The data are not publicly available due to privacy or ethical restrictions.
